# Evaluating different extraction solvents for GC-MS based metabolomic analysis of the fecal metabolome of adult and baby giant pandas

**DOI:** 10.1038/s41598-019-48453-1

**Published:** 2019-08-19

**Authors:** Yang Yang, Yanqiang Yin, Xuyang Chen, Chang Chen, Yinyin Xia, Hongbo Qi, Philip N. Baker, Hua Zhang, Ting-Li Han

**Affiliations:** 1 Department of Obstetrics and Gynecology, The First Affiliated Hospital of Chongqing Medical University, Chongqing, China; 2 Ministry of Education of China International Collaborative Joint Laboratory of Reproduction and Development, Chongqing Medical University, Chongqing, China; 3 State Key Laboratory of Maternal and Fetal Medicine of Chongqing Municipality, Chongqing Medical University, Chongqing, China; 4 Chongqing Zoo, Chongqing, China; 5 Institute of Life Sciences, Chongqing Medical University, Chongqing, China; 6 School of Public Health and Management, Chongqing Medical University, Chongqing, China; 7 College of Life Sciences, University of Leicester, Leicester, UK

**Keywords:** Metabolomics, Zoology

## Abstract

The gut microbiome plays a fundamental role in host health and the fecal metabolome can be analysed to assess microbial activity and can be used as an intermediate phenotype monitoring the host-microbiome relationship. However, there is no established extraction protocol to study the fecal metabolome of giant pandas. The aim of this research is to optimize extraction of the fecal metabolome from adult and baby pandas for high throughput metabolomics analysis using gas chromatography-mass spectrometry (GC-MS). Fecal samples were collected from eight adult pandas and a pair of twin baby pandas. Six different extraction solvents were investigated and evaluated for their reproducibility, metabolite coverage, and extraction efficiency, particularly in relation to the biochemical compound classes such as amino acids, tricarboxylic acid (TCA) cycle intermediates, fatty acids, secondary metabolites, and vitamin and cofactors. Our GC-MS results demonstrated that the extraction solvents with isopropanol: acetonitrile: water (3:2:2 ratio) and 80% methanol were the most appropriate for studying the fecal metabolome of adult and baby giant pandas respectively. These extraction solvents can be used in future study protocols for the analysis of the fecal metabolome in giant pandas.

## Introduction

There is increasing evidence that the gut microbiome plays a fundamental role in host health^1^. Disruption of the gut microbiome contributes to dysregulated homeostasis of host metabolism and is associated with complications including sepsis, inflammatory bowel syndrome^2^, necrotizing enterocolitis^3^, obesity^4^, and diabetes^5^. Metabolomics is the study of a complete set of low-molecular-weight compounds, which make it an ideal omics approach to investigate metabolic activity in the gut. Microbes secrete many metabolites, hormones, and vitamins that can be reabsorbed by the host and affect circulating metabolites such as branched-chain amino acids and trefoil factor 3 which have been shown to be associated with insulin resistance^5^ and bowel syndrome^2^ respectively. The fecal metabolome provides unique information on the metabolic interactions between the gut microbiota, diet, and host. Combined with 16S next-generation-sequencing, the metabolome can provide a comprehensive phenotype of the host-microbiome interplay.

The giant panda (*Ailuropoda melanoleuca*) is one of the most iconic bears in China and is symbolized as a national treasure. Even with this flagship status, the giant panda is a conservation-reliant vulnerable species and hundreds of them are protected in zoos or breeding centers around the world. Female panda often give birth to twin babies. Due to insufficient maternal milk to nourish both cubs, a mixture of breast milk and artificial formulated milk are often delivered. Meanwhile, adult giant pandas feed primarily on bamboo, thus adult panda’s gut microbiota is adapted to a bamboo diet which is optimized to digest cellulose. Although the gut microbiome is known to be closely associated with host health, physiology, and disease, there are no reported studies of the giant panda’s fecal metabolome.

There is no universal strategy for the preparation of fecal material for global metabolite profiling due to the complex nature of its biological matrix and individual biospecimen variation. The reported methodologies are diverse and range from simple to extensive procedures which employ various steps and techniques including homogenisation, lyophilisation, filtration, sonication, centrifugation, solvent extraction, derivatisation, etc^6^. In the metabolic profiling of feces, gas chromatography-mass spectrometry (GC–MS)^7^, nuclear magnetic resonance (NMR) spectroscopy^8^, and liquid chromatography-mass spectrometry (LC-MS)^9^ are the most commonly used analytical platforms. GC-MS is our selected platform because it’s robust and has an advanced separation chromatographic system. The GC-MS spectral fragmentations for compound identification are also highly reproducible. The current practices for fecal sample preparation for GC-MS-based metabolomics typically involve homogenisation before centrifugation, followed by mixing dried fecal material with extraction solvents and derivatisation chemicals. A number of organic solvent compositions have been employed for metabolite extraction, which include different percentages of methanol/water, acetonitrile, a mixture of chloroform/methanol/water, isopropanol/acetonitrile/water, and acetonitrile/chloroform mixtures^10–13^. The majority of published fecal metabolomic studies have investigated human specimens. Considering the panda’s diet and gut microbiota are substantially different to those of humans, an optimal sample preparation method is needed to conduct a reliable panda fecal study.

In this study, we aim to establish a protocol for analyzing the fecal metabolome in both baby and adult giant pandas, which have significantly different dietary intakes. We have established a high throughput fecal preparation pipeline and investigated six different extraction solvents on their reproducibility, metabolite coverage, and extraction efficiency. The protocol resulting from our findings could be used to analyze the fecal metabolome of giant pandas which could in turn serve as a functional reflection of the gut microbiome and be used to further understand the influence of the gut microbiome on giant panda health.

## Results and Discussion

This study was the first to evaluate different extraction solvents to be applied in the process of analyzing the fecal metabolome of adult and baby giant pandas using GC-MS. We have investigated six different extraction solvents that are frequently applied in human fecal metabolome studies. The identified metabolites were subdivided into ten major biochemical classes such as alkanes, amino acids, fatty acids, TCA cycle intermediates, secondary metabolites, and others (Supplementary Tables S1 and S2). Their detailed analytical information is displayed in Supplementary Table S3. Metabolites detected in each of the biochemical classes were assessed in detail to evaluate the performance of the six different extraction solvents on adult and baby panda stools. The performance was evaluated by testing extraction reproducibility, metabolite coverage, and recovery efficiency.

### Analytical qualities of the six different extraction solvents

**Figure 1:**
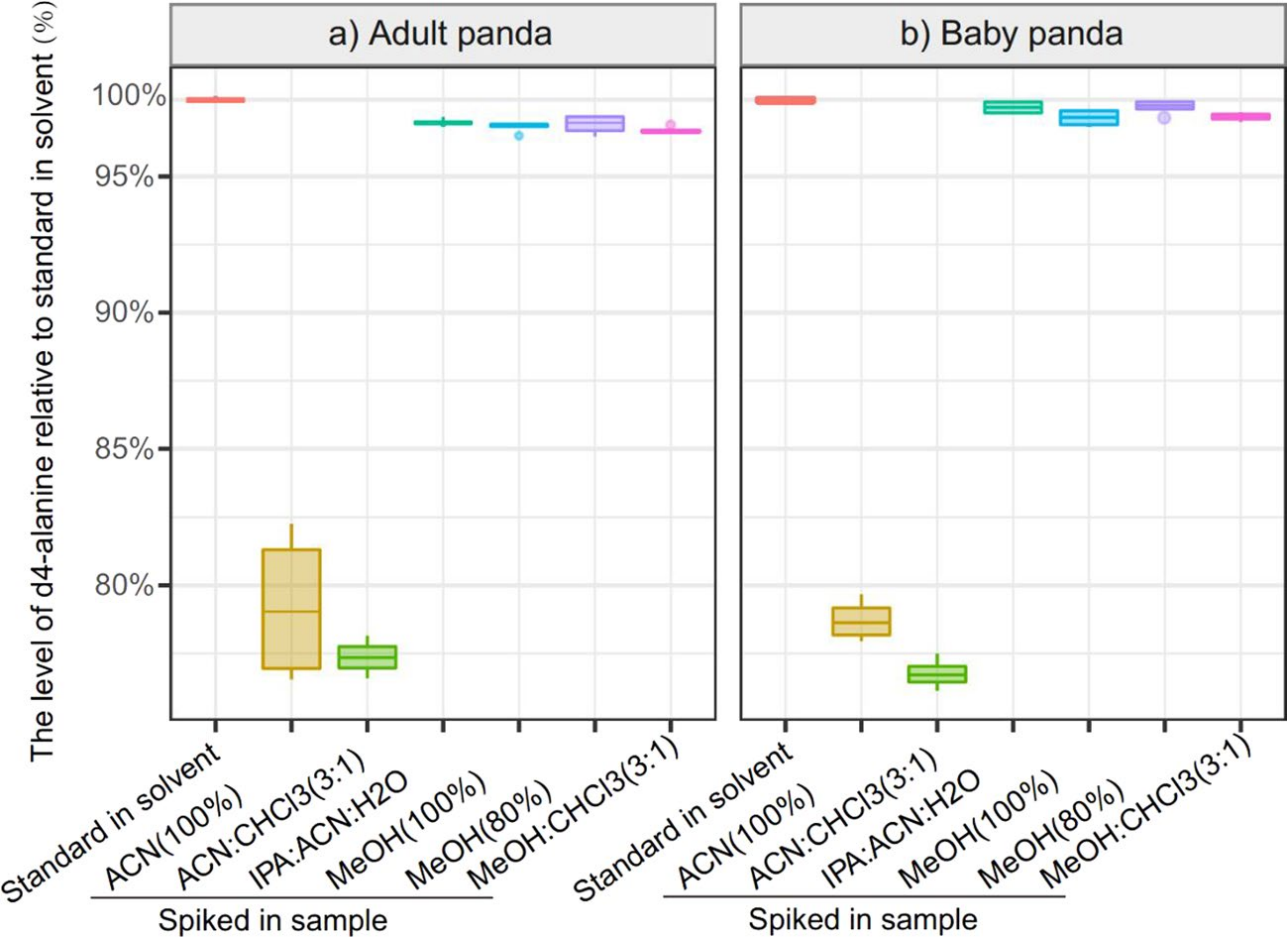
The extraction efficiency of d4-alanine in six different extraction solvents for adult (**a**) and baby (**b**) giant panda feces. d4-alanine was quantified after being spiked directly into the derivatisation chemical (standard in solvent) and levels were compared to d4-alanine levels in the feces samples following different extraction methods (black horizontal lines). The values of d4-alanine were adjusted relative to the levels in the standard in solvent (set to 100%). Four experimental replicates were measured for each extraction method. The distributions of the boxplots are minimum, 25^th^ percentile, median, 75^th^ percentile, and maximum (from bottom to top direction). Dots are outliers (above or below the median by more than 1.5 times the interquartile range).

**Figure 2:**
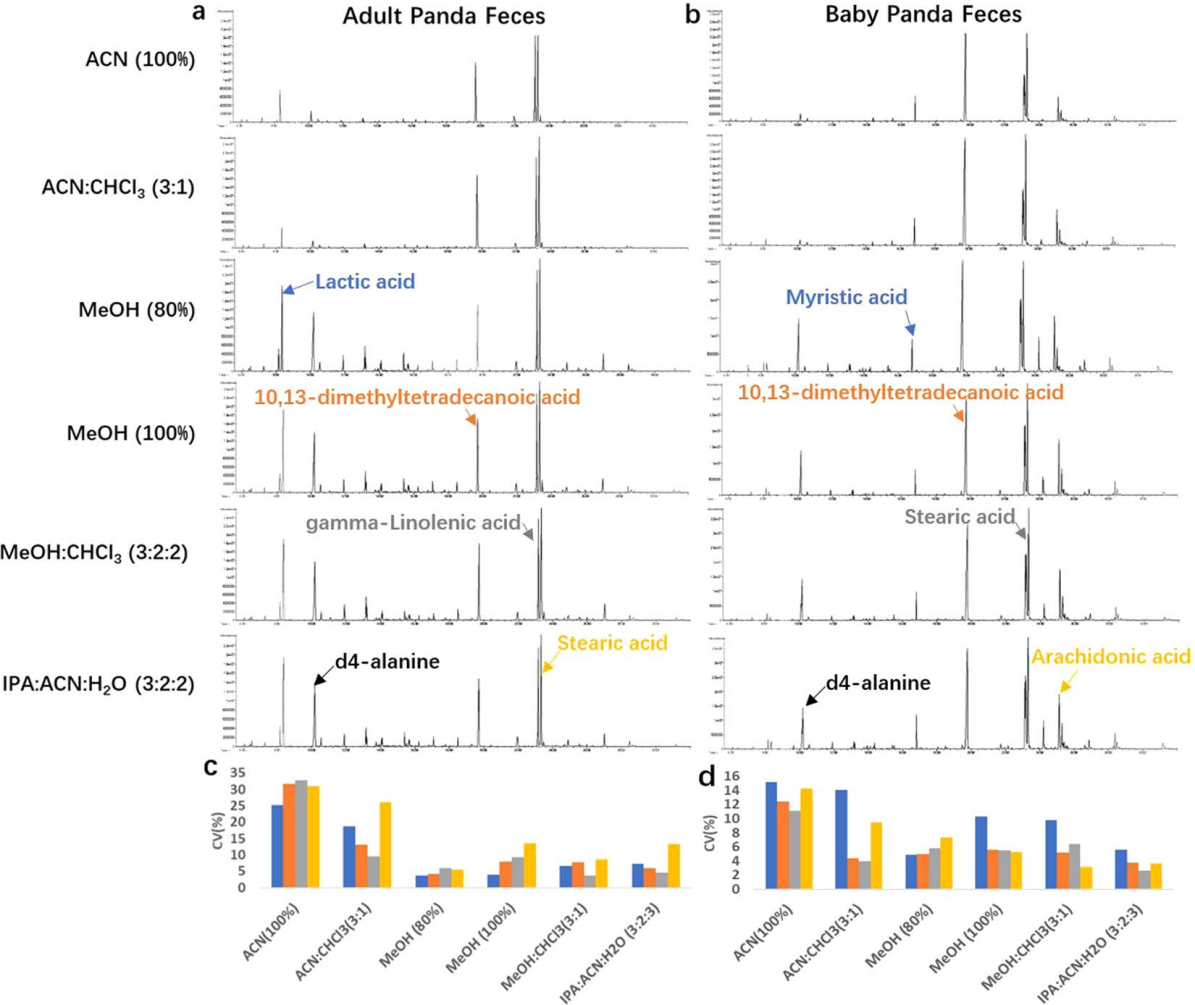
Representative total ion chromatograms (TIC) of the fecal metabolome for adult (**a**) and baby (**b**) giant pandas, using six different exaction solvents. The coefficient of variation (CV) of four highly abundant chromatographic peaks were calculated for adult (**c**: lactic acid, 10,13-dimethyltetradecanoic acid, gamma-linolenic acid, stearic acid) and baby giant pandas (**d**: myristic acid, 10,13-dimethyltetradecanoic acid, stearic acid, arachidonic acid). ACN (100%); acetonitrile: chloroform in 3:1 ratio (ACN:CHCl_3_(3:1)); 80% methanol (MeOH (80%)); 100% methanol (MeOH (100%)); methanol: chloroform in 3:1 ratio (MeOH:CHCl_3_(3:1)); isopropanol: acetonitrile: water in 3:2:2 ratio (APN:ACN:H_2_O (3:2:2)).

The extraction efficiency of six selected methods was first evaluated by comparing the level of d4-alanine standard spiked in a blank solvent (without biological material) to the levels in adult and baby giant panda feces following the different extraction methods (Fig. 1). This result demonstrated that over 95% of d4-alanline was recovered in IPA:ACN:H_2_O, MeOH (100%), MeOH (80%), and MeOH:CHCl_3_ (3:1), while less than 80% of d4-alanine was recovered in ACN(100%) and ACN:CHCl_3_ (3:1). Furthermore, the four highly abundant chromatographic peaks for adult (chosen peaks: lactic acid, 10,13-dimethyltetradecanoic acid, gamma-linolenic acid, stearic acid) and baby pandas (chosen peaks: myristic acid, 10,13-dimethyltetradecanoic acid, stearic acid, arachidonic acid) were selected to assess the extraction reproducibility. MeOH (80%) showed the lowest variation in these abundant peaks for the adult panda (Fig. 2c), whilst IPA:ACN:H_2_0 had the best reproducibility for the baby panda feces (Fig. 2d).

### Overall GC-chromatogram assessment

The representative total ion chromatograms (TIC) for the fecal extracts resulting from the six different extraction solvents tested in this study are illustrated in Fig. 2. We observed discrepancies in the distribution of chromatographic peaks between the adult and baby panda fecal metabolomes. There was also a fewer number of peaks in the chromatogram following extraction with acetonitrile constituted solvents including ACN (100%) and ACN:CHCl_3_ (3:1), for both adult and baby panda stools.

After chromatographic peak deconvolution and identification, over 200 peaks were detected and 120 metabolites were identified using our in-house MS library of chemical standards. Of the metabolites identified, we classified 75 and 82 metabolites into ten biochemical groups, for adult and baby panda stools respectively. There was a significantly lower number of metabolites, particularly long-chain fatty acids, found in the adult panda stools compared to the baby panda (Supplementary Tables S1 and S2). It is likely because the adult pandas are fed exclusively on bamboo shoots/leaves which have low nutritional content compared to breast milk/formulated milk fed to the baby pandas^14^.

### Reproducibility of the six different extraction solvents for adult and baby panda stools

**Figure 3:**
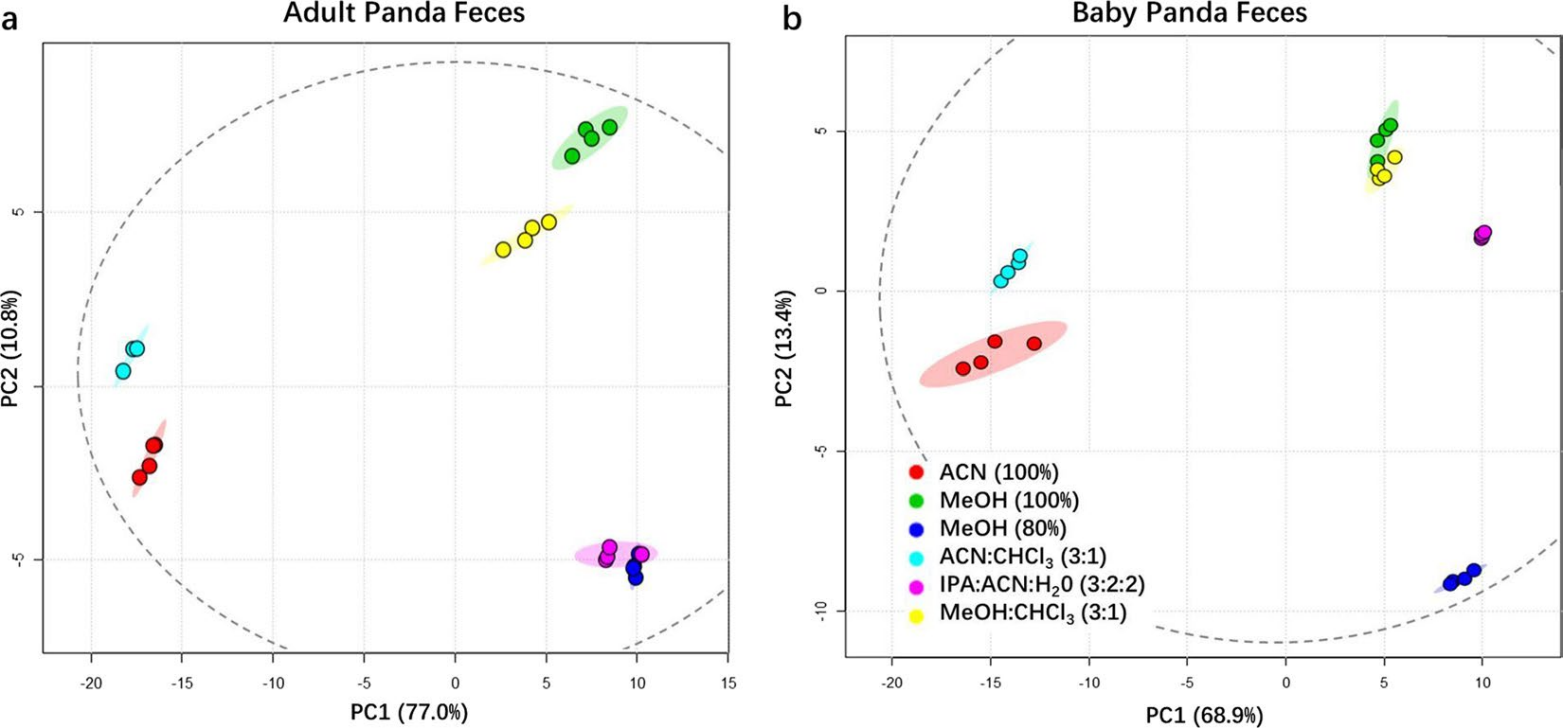
Principal component analysis (PCA) of the adult (**a**) and baby (**b**) giant panda fecal metabolomes, extracted using six different solvents. Each color represents an extraction method. Red colored dots represent ACN (100%); green colored dots represent MeOH (100%); blue colored dots represent MeOH (80%); cyan colored dots represent ACN:CHCl_3_ (3:1); Purple dots represent IPA:ACN:H_2_O (3:2:2); and yellow dots represent MeOH:CHCl_3_ (3:1). An ellipse with dotted lines indicates the 95% confidence interval calculated by Hotelling’s T2 statistics.

**Figure 4:**
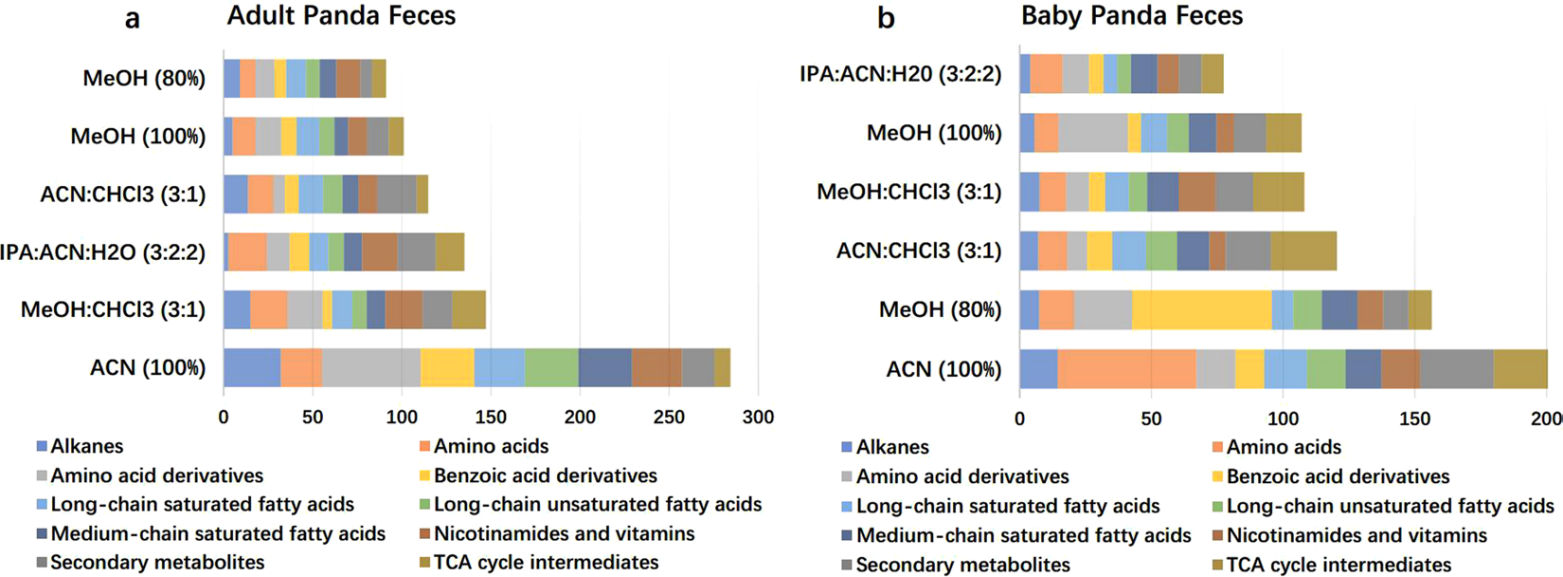
The bar chart represents the cumulative coefficient of variations (CV) of ten different biochemical classes using six different extraction solvents. (**a**) CVs of the fecal metabolome from adult giant panda. (**b**) CVs of the fecal metabolome from baby giant panda. The major metabolite classifications are alkanes, amino acids, amino acid derivatives, benzoic acids and derivatives, long-chain saturated fatty acids, long-chain unsaturated fatty acids, medium-chain saturated fatty acids, nicotinamides and vitamins, and secondary metabolites, and TCA cycle intermediates. The x-axis is the total CV for all major metabolite classes.

MeOH (80%) and IPA:ACN:H_2_0 (3:2:2) showed the most promising extraction reproducibility for adult and baby giant pandas respectively. The PCA analysis demonstrated the technical replicates of MeOH (80%) fecal extracts (blue dots) were clustered the closest to each other along PC1 (77%) and PC2 (10.8%) for adult panda **(**Fig. 3a**)**, while technical replicates of IPA:ACN:H_2_O (3:2:2) fecal extracts (purple dots) were clustered the closest to each other along PC1 (68.9%) and PC2 (13.4%) for baby panda **(**Fig. 3b**)**. Consistent with the results of the PCA, MeOH (80%) and IPA:ACN:H_2_0 (3:2:2) exhibited the most reproducible coefficient of variances (CV: 9.8%, 8.7%) for the overall biochemical classes identified in adult and baby panda stools respectively **(**Supplementary Tables S1, S2 and Fig. 4). Importantly, ACN (100%) displayed the least reproducible CVs for both adult (CV: 27.5%) and baby (CV: 25.4%) panda stools. Interestingly, the reproducability of IPA:ACN:H_2_O (3:2:2) for baby panda stools was exceptional for unsaturated long-chain fatty acids (CV: 5.13%) and saturated long-chain fatty acids (CV: 5.23%) but its CV for amino acids was inferior compared to other extraction solvents except ACN (100%). Therefore, IPA:ACN:H_2_O (3:2:2) is an ideal extraction solvent for fatty acids but not ideal for amino acid extraction. The possible underlying reason that IPA:ACN:H_2_O (3:2:2) possesses superior reproducibility for extracting fatty acids could be the fact that isopropanol is an inhibitor of phospholipase D and thus protects lipids in their native form^15^.

### Metabolite coverage of the six different extraction solvents for adult and baby panda stools

**Figure 5:**
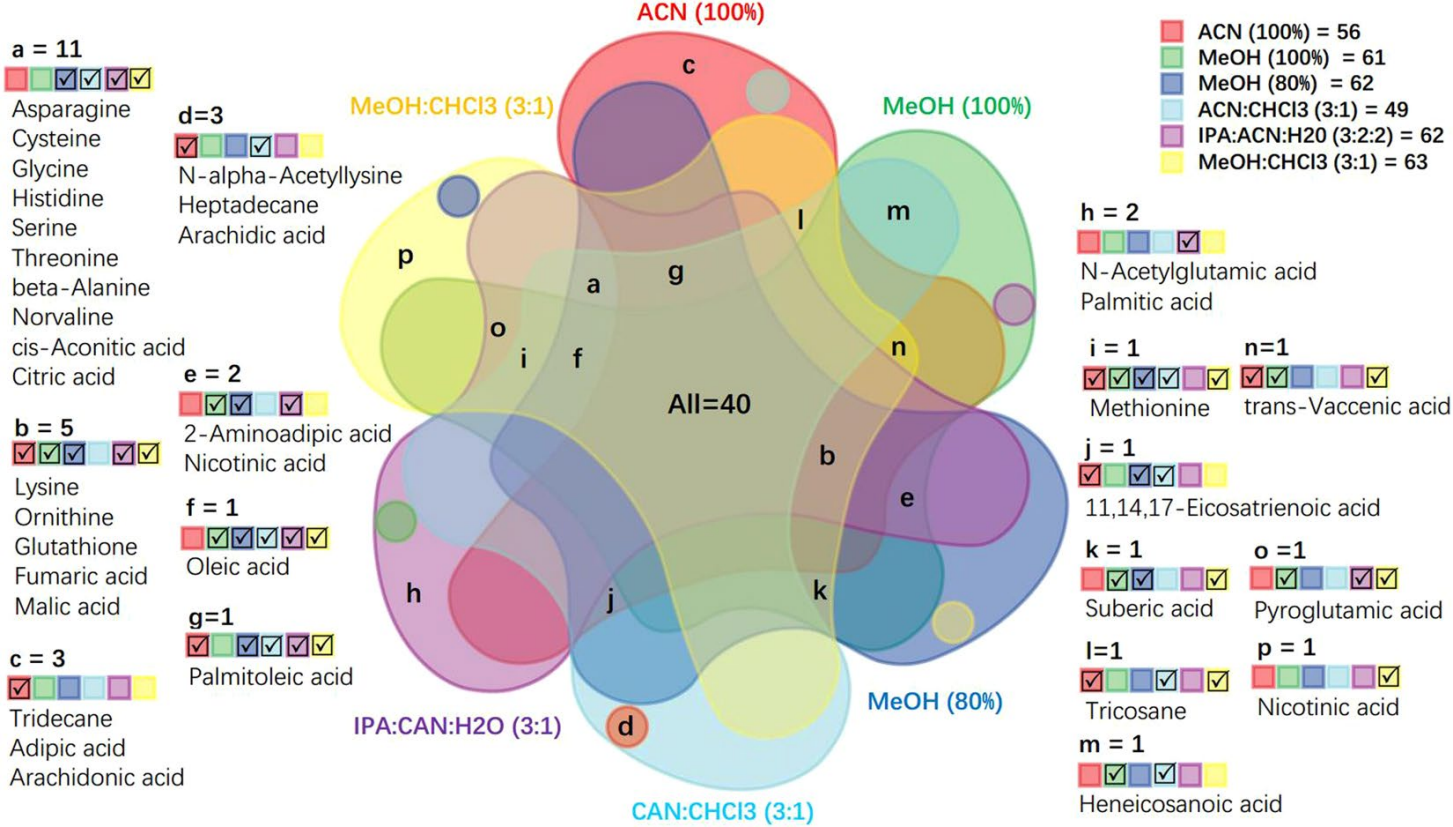
Six-way Venn visualization of the identified metabolite coverage compared across six different extraction methods, in adult panda feces. Red: ACN (100%); green: MeOH (100%); blue: MeOH (80%); cyan: ACN:CHCl_3_ (3:1); purple: IPA:ACN:H_2_O (3:2:2); yellow: MeOH:CHCl_3_ (3:1). Letter = number of identified metabolites in each color region. √ the existence of extraction methods in the overlapped region.

**Figure 6:**
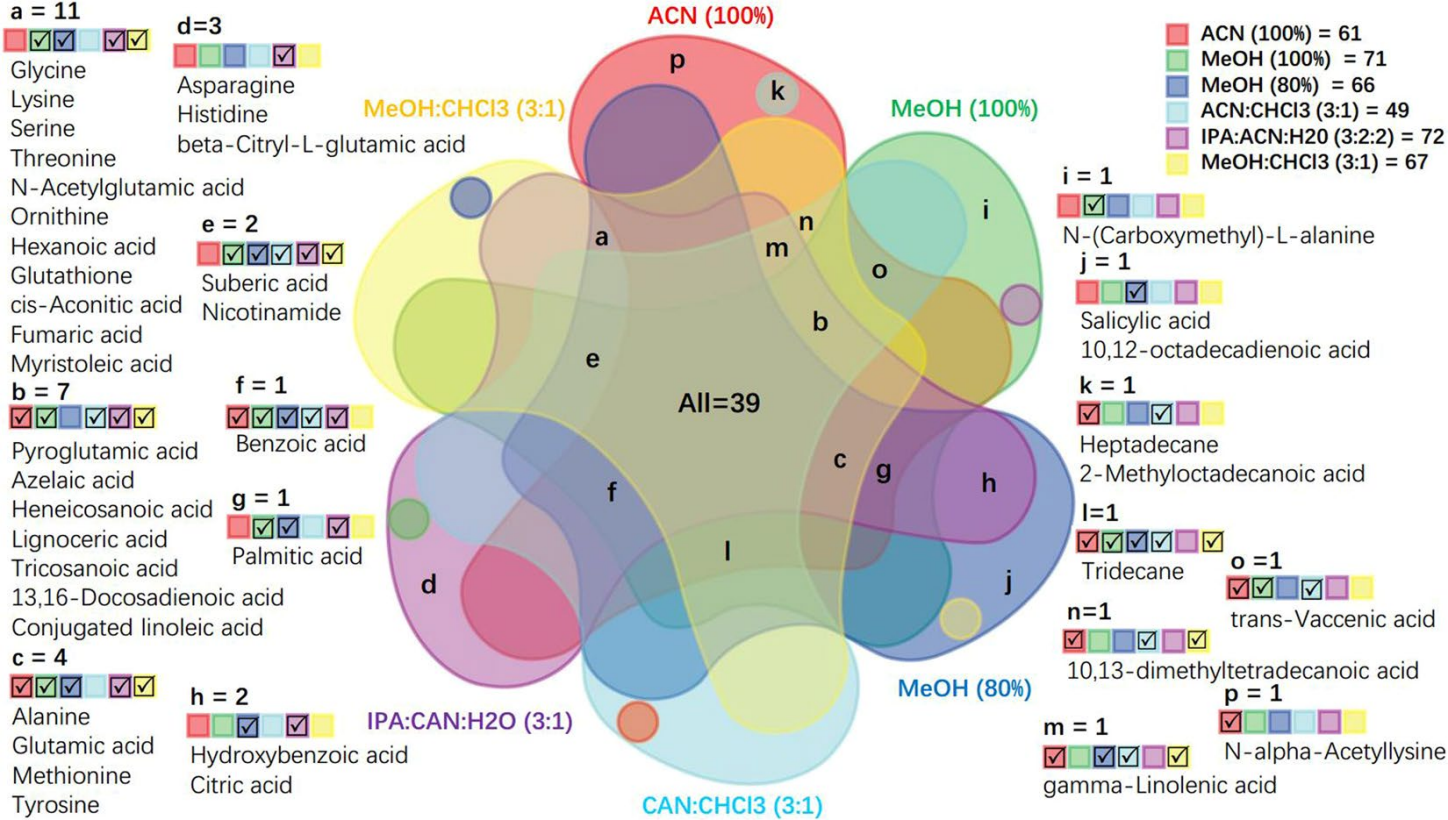
Six-way Venn visualization of the identified metabolite coverage compared across six different extraction methods, in baby panda feces. Red: ACN (100%); green: MeOH (100%); blue: MeOH (80%); cyan: ACN:CHCl_3_ (3:1); purple: IPA:ACN:H_2_O (3:2:2); yellow: MeOH:CHCl_3_ (3:1). Letter = number of identified metabolites in each color region. √ the existence of extraction methods in the overlapped region.

The second criterion we used to determine the optimal extraction solvent was the metabolite coverage in the fecal metabolome of adult and baby giant pandas. The order of extraction solvents producing the highest to lowest number of identified metabolites from adult panda stools (Supplementary Tables S1 and S4) were: MeOH (80%; 110 metabolites), IPA:ACN:H_2_O (3:2:2; 108 metabolites), MeOH (100%; 104 metabolites), MeOH:CHCl_3_ (3:1; 100 metabolites), ACN (100%; 84 metabolites), and ACN:CHCl_3_(3:1: 73 metabolites). The ranking of extraction solvents metabolite coverage of baby panda stool (Supplementary Tables S2 and S5) were: IPA:ACN:H_2_O (3:2:2; 120 metabolites), MeOH (80%; 107 metabolites), MeOH (100%; 106 metabolites), MeOH:CHCl_3_ (3:1; 102 metabolites), ACN (100%; 85 metabolites), and ACN:CHCl_3_ (3:1; 79 metabolites). Furthermore, the six-way Venn diagram illustrated that the methanol-based solvents (MeOH (80%) and MeOH:CHCl_3_ (3:1)) had the highest overlap in metabolite coverage for adult panda (Fig. 5: All(40) + a(11) + b(5) + f (1) + g (1) + i(1) + k(1) = 60), and IPA:ACN:H_2_O (3:2:2) and MeOH (100%) had the highest overlap in metabolite coverage for baby panda (Fig. 6: All(39) + a(11) + b(7) + c(4) + e(2) + f(1) + g(1) = 65). Overall, MeOH (80%) and IPA:ACN:H_2_O (3:2:2) showed the most comprehensive coverage of total identified metabolites for the adult and baby pandas respectively.

### Extraction recovery efficiency of the six different extraction solvents for adult and baby panda stools

**Figure 7:**
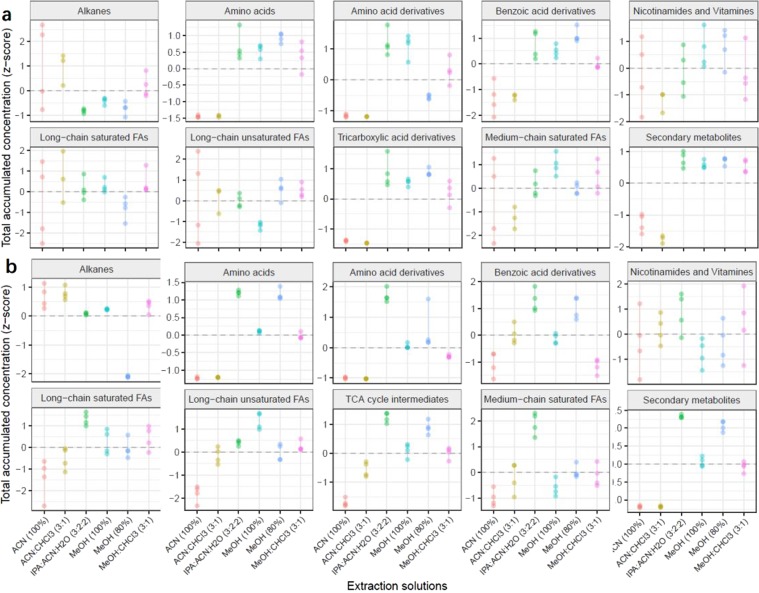
The dot-line graphs show the total concentrations of metabolites in each metabolic biochemical class across six different extraction solvents for adult (**a**) and baby (**b**) giant panda feces. Each dot represents a sample concentration. Red dots: ACN (100%); green dots: MeOH (100%); blue dots: MeOH (80%); cyan dots: ACN:CHCl_3_ (3:1); purple dots: IPA:ACN:H_2_O (3:2:2); yellow dots: MeOH:CHCl_3_ (3:1). The vertical lines are the standard deviation of each extraction method. The metabolite concentrations were standardized to Z-scores.

The last criterion for the evaluation of the extraction solvents is extraction efficiency, which is determined by the sum of all peak intensities of extracted metabolites in a similar biochemical class, as shown by Fig. 7 and Supplementary Tables S1 and S2. For the adult panda fecal metabolome, methanol (80%) displayed the highest peak intensity for amino acids, benzoic acid derivatives, long-chain unsaturated fatty acids, and TCA cycle intermediates; IPA:ACN:H_2_O (3:2:2) produced the greatest recovery yield for amino acid derivatives and secondary metabolites; MeOH (100%) was best for nicotinamides/vitamins and long-chain unsaturated fatty acids; and ACN (100%) was superior for alkanes. Conversely, in the case of the baby panda fecal metabolome, IPA:ACN:H_2_O (3:2:2) not only demonstrated the highest peak intensity for all the biochemical classes involved in the central carbon metabolism, but also the greatest recovery yield for long-chain saturated fatty acids and medium-chain saturated fatty acids with the only exception being medium-chain fatty acids (100% methanol was optimal) and alkanes. Worthy of noting, ACN (100%) and ACN:Cl_3_ (3:1) produced the highest peak intensity for the alkane class yet the lowest peak intensity for the chemical classes of amino acids, amino acid derivatives, benzoic acids, TCA cycle intermediates, and secondary metabolites, in both adult and baby panda stools. Since these two extraction solvents are non-polar solvents, they are efficient extraction solvents for the non-polar compounds such as alkanes but ineffective at extracting polar compounds such as amino acids and TCA cycle intermediates.

In summary, our findings suggest that out of the six extraction solvents tested, MeOH (80%) had the most optimal extraction efficiency for extracting metabolites from adult panda stool, and IPA:ACN:H_2_O (3:2:2) for extracting metabolites from baby panda stool.

In conclusion, this is the first study conducted to investigate the optimal extraction solvent for analyzing the global fecal metabolome of giant pandas by GC-MS analysis. Based on the criteria of reproducibility of extraction, detected metabolite coverage, and the extraction recovery yield, MeOH (80%) and IPA:ACN:H_2_0 (3:2:2) were the superior extraction solvents for adult and baby giant panda respectively. However, different organic solvents favor the extraction of specific biochemical classes. Future studies need to carefully select their extraction solvent to meet the primary purpose of their fecal metabolome study.

## Methods

### Panda feces collections

Giant pandas and twin babies were raised at the Chongqing Zoo in China. We collected the fecal samples from eight adult pandas once and one-month-old twin panda babies eight times. The fresh fecal samples were transferred into sterile tubes and stored in a −80 °C freezer immediately. Fecal samples were collected by certified zoo staff and the process was approved by the Chongqing Zoo Ethics Committee.

### Fecal metabolome extraction solvents

Six different extraction solvents were chosen based on pre-exising protocols for the analysis of the human fecal metabolome. The compositions of each extraction solvent were: 100% acetonitrile (ACN (100%))^16^; acetonitrile: chloroform in 3:1 ratio (ACN: CHCl_3_(3:1)); 80% methanol (MeOH (80%))^17,18^: 100% methanol (MeOH (100%))^12,19^; methanol: chloroform in 3:1 ratio (MeOH:CHCl_3_(3:1))^10^; and isopropanol: acetonitrile: water in 3:2:2 ratio (APN:ACN:H_2_O (3:2:2))^11^. An internal standard, L-alanine-2,3,3,3-d4 (10 mM), was added into each extraction solvent.

### Sample preparation for panda feces

All fecal samples were dried in a SpeedVac (Labconco^TM^) for 5 h and all of them were mixed together in a pestle and mortar to ensure homogeneity of the stools for all tested extraction methods. Four replicates of 10 mg ± 1 mg of mixed stool were weighed into 2 mL screw-cap tubes, for each tested solvent. Subsequently, 600 ul of the extraction solvent – internal standard mix was added. Metal beads were transferred into each screw-cap tube and the stool-solvent mix was homogenized using a Qiagen Tissuelyser-II at 30 Hz for 3 min. After centrifugation at 17000 g for 15 min the supernatants were isolated and dried by SpeedVac for 3 h. Dried fecal extracts were stored in a −80 °C freezer until derivatization.

### Methyl chloroformate derivatization

The extracted fecal metabolites were derivatized using a methyl chloroformate (MCF) approach based on the protocol published by Smart *et al*.^20^. MCF derivatisation was chosen because its reaction can take place in the aqueous sample and is less prone to matrix interference, making it suitable for derivatisation of semi-dried feces containing complex matrix-associated compounds including proteins, lipids, water, and sugars. In brief, 200 µl of sodium hydroxide (1 M) was added to the dried samples, followed by 167 µL of methanol and 34 µL of pyridine. Subsequently, 20 µL MCF was added, followed by 30 s of vortexing, and another 20 µL addition of MCF followed by 30 s of vortexing. 400 µL of chloroform and 400 µL of sodium bicarbonate (50 mM) were added and vortexed for 10 s to isolate derivatized metabolites from the reactive mixture. The resulting lower chloroform phase was isolated for GC-MS analysis.

### Gas chromatography-mass spectrometry (GC-MS) analysis

The derivatized samples were analyzed using an Agilent GC7890 system linked to a MSD5975 with electron impact ionization (70 eV). The gas capillary column was a ZB-1701 (30 m × 250 μm id × 0.15 μm with 5 m guard column, Phenomenex). The parameters of the GC oven and MS were operated in accordance with Smart *et al*.^20^’s published protocol. The samples were injected into a pulsed splitless mode inlet at 290 °C with the flow of helium gas at 1 mL.min^−1^. The temperatures of the auxiliary, MS quadrupole, and MS source were 250 °C, 230 °C, and 150 °C respectively. The mass range was detected from 30 um to 550 µm. Scan speed was set to 1.562 µ.s^−1^ and the solvent delay was applied until 5.5 min. The estimated time required for sample preparation, derivatisation, and GC-MS acquisition is shown in Supplementary Table S6.

### Metabolite identification, data mining, normalization, and statistical analysis

Metabolite deconvolutions and identifications were performed using AMDIS software and our in-house MCF mass spectral library. After GC peaks were deconvoluted and background subtraction performed, the compounds were identified according to the following two criteria: 1) >85% library match factor that was calculated by the similarity of the extracted spectrum and library spectra via match pure:impure spectra combined linearly in a 7:3 ratio; 2) within a one-minute window of the respective GC retention time, extra time has been included to cover the retention time shift resulting from the effect of the different biological matrices and GC column trimming. The chromatograph height (relative concentration) of the reference ion for each metabolite was extracted using our in-house MassOmics R-based package^20^. The relative metabolite concentrations were normalized by the concentration of the internal standard d4-alanine and the dried-weight of the fecal sample. Statistical analyses were performed using SPSS version 24.0 software and Microsoft Excel. Principal component analysis (PCA) and coefficient of variance (CV) were used to check the repeatability of extraction methods. Two-dimensional projections of principal component analysis (PCA) and dot plots were rendered using the ggplot2 R-based package^21^. Metabolite coverage was compared across the six extraction protocols and demonstrated using Venn diagrams, which were generated using the VennDiagram R package^22^ and were subsequently illustrated in Adobe Illustrator.

## Supplementary information

Supplementary Information

## Data Availability

The data that support the findings of this study are available from the corresponding author upon reasonable request.
