# Supplementary material for: Evaluating different extraction solvents for GC-MS based metabolomic analysis of the fecal metabolome of adult and baby giant pandas
Source: Sci Rep. 2019 Aug 19;9:12017. doi: 10.1038/s41598-019-48453-1 (PMC6700143; doi:10.1038/s41598-019-48453-1)
Supplement: Supplementary file 1 — Supplementary Information [file 41598_2019_48453_MOESM1_ESM.pdf]

# Evaluating different extraction solvents for GC-MS based metabolomic analysis of the fecal metabolome of adult and baby giant pandas

**Yang yang[1,2,3], Yanqiang Yin[4], Xuyang Chen[1], Chang Chen[1,5], Yinyin Xia[6], Hongbo Qi[1,2,3], Philip N. Baker[1,7], Hua Zhang[1,2,3]*, Ting-Li Han[1,2,3,*]**

**Supplementary table S1** Number of identified metabolites and CVs of each biochemical compound class identified in adult giant panda feces, compared across six different extraction methods

| Biochemical Classes | IPA:ACN:H2O (3:2:2) | | MeOH (100%) | | MeOH (80%) | | MeOH:CHCl3(3:1) | | ACN(100%) | | ACN:CHCl3(3:1) | |
|---|---|---|---|---|---|---|---|---|---|---|---|---|
| | # | CV±SD | # | CV±SD | # | CV±SD | # | CV±SD | # | CV±SD | # | CV±SD |
| Alkanes | 2 | 2.6±0.1 | 2 | 5.0±2.4 | 2 | 9.1±3.2 | 3 | 14.9±14.8 | 5 | 32.0±1.7 | 4 | 13.5±3.9 |
| Amino acids | 17 | 21.5±5.8 | 18 | 12.8±4.6 | 18 | 8.7±3.7 | 18 | 20.8±5.3 | 12 | 23.1±10.6 | 11 | 14.5±3.0 |
| Amino acid derivatives | 5 | 12.9±7.2 | 4 | 14.5±5.8 | 3 | 10.6±2.8 | 4 | 19.8±7.7 | 2 | 55.5±44.9 | 1 | 6.3 |
| Benzoic acid derivatives | 6 | 11.0±5.5 | 6 | 8.5±3.6 | 6 | 6.6±2.5 | 6 | 5.3±1.6 | 6 | 30.1±4.4 | 6 | 7.8±3.7 |
| Nicotinamides and vitamines | 2 | 19.7±8.6 | 2 | 10.5±6.5 | 2 | 13.4±3.3 | 1 | 21 | 1 | 28.0± | 1 | 11 |
| Long-chain saturated fatty acids | 9 | 10.7±3.8 | 9 | 12.8±9.7 | 8 | 11.2±6.3 | 8 | 11.3±4.9 | 10 | 28.3±3.5 | 10 | 13.7±4.9 |
| Long-chain unsaturated fatty acids | 7 | 8.7±5.8 | 5 | 8.5±6.0 | 8 | 7.6±3.2 | 9 | 8.1±4.3 | 8 | 30.0±2.8 | 8 | 10.8±2.2 |
| TCA cycle intermediates | 5 | 16.1±5.6 | 5 | 8.3±5.5 | 5 | 8.0±4.2 | 5 | 18.8±7.4 | 3 | 9.0±5.7 | 1 | 6.4 |
| Medium-chain saturated fatty acids | 6 | 10.3±4.8 | 7 | 7.8±3.8 | 7 | 9.3±3.1 | 6 | 10.6±6.7 | 6 | 30.2±4.7 | 5 | 9.1±1.1 |
| Secondary metabolites | 3 | 21.6±18.3 | 3 | 12.3±10.1 | 3 | 10.1±6.8 | 3 | 16.8±9.8 | 3 | 18.2±6.0 | 2 | 22.2±5.0 |
| Sum of classified metabolites | 62 | 15.1±8.7 | 61 | 11.4±6.6 | 62 | 9.8±4.8 | 63 | 14.2±7.8 | 56 | 27.5±10.9 | 49 | 11.8±4.8 |
| Total identified metabolits | 108 | 16.2±10.9 | 104 | 12.9±9.2 | 110 | 10.6±6.0 | 100 | 14.1±8.0 | 84 | 26.8±10.5 | 73 | 13.1±6.1 |

#: Number of identified metabolites; CV: coefficient of variance in %; SD: standard deviations in %

**Supplementary table S2.** Number of identified metabolites and CVs of each biochemical compound class identified in baby giant panda feces, compared across six different extraction methods

| Biochemical Classes | IPA:ACN:H2O (3:2:2) | | MeOH (100%) | | MeOH (80%) | | MeOH:CHCl3 (3:1) | | ACN (100%) | | ACN:CHCl3 (3:1) | |
|---|---|---|---|---|---|---|---|---|---|---|---|---|
| | # | CV±SD | # | CV±SD | # | CV±SD | # | CV±SD | # | CV±SD | # | CV±SD |
| Alkanes | 2 | 4.0±0.8 | 3 | 5.6±5.3 | 3 | 7.4±1.4 | 3 | 7.5±0.8 | 4 | 14.4±6.0 | 4 | 7.0±1.5 |
| Amino acids | 18 | 12.3±14.1 | 16 | 9.1±7.6 | 17 | 13.3±9.0 | 16 | 10.1±4.6 | 12 | 52.7±30.3 | 8 | 11.1±8.2 |
| Amino acid derivatives | 5 | 10.0±7.6 | 6 | 26.37±26.4 | 3 | 22.0±23.9 | 4 | 8.7±2.3 | 3 | 14.8±7.0 | 2 | 7.5±2.0 |
| Benzoic acid derivatives | 3 | 5.5±3.2 | 2 | 5.0±0.8 | 3 | 8.7±2.6 | 1 | 6 | 2 | 10.9±0.6 | 2 | 9.5±5.2 |
| Nicotinamides and Vitamines | 1 | 8 | 1 | 7 | 1 | 10 | 1 | 14 | 1 | 15 | 1 | 6 |
| Long-chain saturated fatty acids | 12 | 5.28±2.01 | 12 | 10.0±3.8 | 9 | 8.1±4.5 | 12 | 8.9±2.9 | 13 | 16.2±6.5 | 13 | 12.1±7.4 |
| Long-chain unsaturated fatty acids | 15 | 5.13±3.67 | 16 | 8.1±3.6 | 15 | 10.9±5.3 | 15 | 6.9±3.2 | 16 | 14.7±1.9 | 16 | 11.8±4.1 |
| TCA cycle intermediates | 6 | 8.29±2.4 | 5 | 13.5±3.2 | 6 | 8.8±3.9 | 5 | 19.5±21.4 | 3 | 20.8±4.5 | 3 | 25.0±17.1 |
| Medium-chain saturated fatty acids | 7 | 10.1±2.7 | 7 | 10.5±4.7 | 6 | 13.6±7.2 | 7 | 12.1±5.0 | 5 | 13.2±4.5 | 6 | 12.3±5.6 |
| Secondary metabolites | 3 | 8.7±7.7 | 3 | 12.2±3.4 | 3 | 9.6±8.9 | 3 | 14.2±3.0 | 2 | 28.1±2.0 | 2 | 17.2±7.1 |
| Sum of classifed metabolites | 72 | 8.7±7.8 | 71 | 10.9±10.0 | 66 | 10.9±7.6 | 67 | 10.5±7.8 | 61 | 24.5±26.3 | 57 | 11.9±6.6 |
| Total identified metabolites | 120 | 10.0±8.5 | 106 | 12.0±12.2 | 107 | 12.6±9.7 | 102 | 11.7±11.4 | 85 | 25.3±26.1 | 79 | 12.6±8.0 |

#: Number of identified metabolites; CV: coefficient of variance in %; SD: standard deviations in %

**Supplementary Table S3**. The analytical information of identified metabolites in the giant panda feces

| Name | Classification | Reference Ion | Retention time (min) | Library match | Peak width |
|---|---|---|---|---|---|
| Dodecane | Alkanes | 57 | 8.757 | 99 | 4.8 |
| Heptadecane | Alkanes | 57 | 15.662 | 81 | 5.5 |
| Pentadecane | Alkanes | 57 | 13.131 | 90 | 5.4 |
| Tricosane | Alkanes | 57 | 24.276 | 81 | 4 |
| Tridecane | Alkanes | 57 | 10.303 | 93 | 6.6 |
| Alanine | Amino acids | 102 | 10.209 | 96 | 2.7 |
| Asparagine | Amino acids | 127 | 15.606 | 75 | 4.5 |
| Aspartic acid | Amino acids | 160 | 15.402 | 99 | 3.95 |
| Cysteine | Amino acids | 192 | 18.605 | 96 | 5.45 |
| Glutamic acid | Amino acids | 174 | 17.081 | 95 | 4.15 |
| Glycine | Amino acids | 88 | 10.548 | 82 | 4.2 |
| Histidine | Amino acids | 139 | 25.568 | 89 | 5.9 |
| Isoleucine | Amino acids | 115 | 13.202 | 99 | 3.95 |
| Leucine | Amino acids | 144 | 13.155 | 98 | 4 |
| Lysine | Amino acids | 142 | 24.908 | 86 | 5.75 |
| Methionine | Amino acids | 147 | 17.134 | 86 | 4.45 |
| Phenylalanine | Amino acids | 162 | 18.499 | 99 | 5.45 |

| | | | | | |
|---|---|---|---|---|---|
| Proline | Amino acids | 128 | 14.105 | 99 | 4.3 |
| Serine | Amino acids | 100 | 16.443 | 83 | 4.1 |
| Threonine | Amino acids | 115 | 14.714 | 98 | 5.75 |
| Tryptophan | Amino acids | 130 | 31.186 | 99 | 8.95 |
| Tyrosine | Amino acids | 236 | 27.006 | 99 | 7.35 |
| Valine | Amino acids | 130 | 11.9 | 100 | 3.9 |
| beta-Alanine | Amino acids and derivatives | 88 | 11.801 | 96 | 4.5 |
| beta-Citryl-L-glutamic acid | Amino acids and derivatives | 143 | 29.923 | 75 | 8.9 |
| N-(Carboxymethyl)-L-alanine | Amino acids and derivatives | 174 | 14.482 | 75 | 5.1 |
| N-Acetylglutamic acid | Amino acids and derivatives | 116 | 17.895 | 76 | 4.9 |
| N-alpha-Acetyllysine | Amino acids and derivatives | 129 | 27.7 | 91 | 8.2 |
| Ornithine | Amino acids and derivatives | 128 | 23.703 | 94 | 4.45 |
| Pyroglutamic acid | Amino acids and derivatives | 84 | 15.415 | 90 | 5.3 |
| Benzoic acid | Benzoic acids and derivatives | 105 | 8.711 | 98 | 6.05 |
| DBP | Benzoic acids and derivatives | 149 | 22.774 | 86 | 4.2 |
| Hydroxybenzoic acid | Benzoic acids and derivatives | 135 | 16.23 | 97 | 4.05 |
| Salicylic acid | Benzoic acids and derivatives | 135 | 15.873 | 87 | 4.15 |
| 4-Methyl-2-oxopentanoic acid | Keto acids and derivatives | 85 | 7.021 | 94 | 4.1 |
| Levulinic acid | Keto acids and derivatives | 99 | 8.218 | 75 | 5 |
| 2-Aminoadipic acid | Medium-chain fatty acids | 114 | 18.568 | 83 | 5.2 |
| Adipic acid | Medium-chain fatty acids | 114 | 11.505 | 85 | 6.15 |
| Azelaic acid | Medium-chain fatty acids | 185 | 15.585 | 82 | 4.2 |
| Caprinoic acid | Medium-chain fatty acids | 74 | 11.654 | 92 | 4.5 |
| Decanoic acid (C10_0) | Medium-chain fatty acids | 74 | 11.656 | 88 | 5.1 |
| Dodecanoic acid (C12_0) | Medium-chain fatty acids | 74 | 14.366 | 98 | 3.9 |
| Hexanoic acid (C6_0) | Medium-chain fatty acids | 74 | 8.223 | 75 | 4.2 |
| Octanoic acid (C8_0) | Medium-chain fatty acids | 74 | 8.616 | 84 | 5.55 |
| Suberic acid | Medium-chain fatty acids | 129 | 14.312 | 92 | 4.15 |
| Nicotinamide | Nicotinamides and Vitamines | 57 | 7.172 | 77 | 5.8 |
| Nicotinic acid | Nicotinamides and Vitamines | 137 | 9.852 | 78 | 6.05 |
| 10,13-dimethyltetradecanoic acid (C17_0) | Saturated fatty acids | 74 | 19.746 | 98 | 14 |
| 2-Methyloctadecanoic acid | Saturated fatty acids | 88 | 23.939 | 76 | 4.9 |

| | | | | | |
|---|---|---|---|---|---|
| Arachidic acid (C20_0) | Saturated fatty acids | 74 | 25.558 | 89 | 5.75 |
| Arachidonic acid (C20_4n-6,9,12,15c) | Saturated fatty acids | 79 | 25.125 | 98 | 6.15 |
| Behenic acid (C22_0) | Saturated fatty acids | 74 | 28.679 | 85 | 5.55 |
| Heneicosanoic acid (C21_0) | Saturated fatty acids | 74 | 27.068 | 70 | 7.1 |
| Lignoceric acid (C24_0) | Saturated fatty acids | 74 | 31.479 | 88 | 8.55 |
| Margaric acid (C17_0) | Saturated fatty acids | 74 | 21.938 | 96 | 9.05 |
| Myristic acid (C14_0) | Saturated fatty acids | 74 | 16.81 | 98 | 3.95 |
| Nonadecanoic acid (C19_0) | Saturated fatty acids | 74 | 24.328 | 75 | 4.95 |
| Palmitic acid (C16_0) | Saturated fatty acids | 74 | 19.756 | 81 | 9 |
| Pentadecanoic acid (C15_0) | Saturated fatty acids | 74 | 18.056 | 84 | 4.9 |
| Stearic acid (C18_0) | Saturated fatty acids | 74 | 23.308 | 76 | 7 |
| Tricosanoic acid (C23_0) | Saturated fatty acids | 74 | 29.944 | 81 | 7.45 |
| Creatinine | Secondary metabolites | 202 | 15.83 | 96 | 3.9 |
| Glutathione | secondary metabolites | 142 | 17.631 | 91 | 4.9 |
| Lactic acid | secondary metabolites | 103 | 8.328 | 99 | 4.4 |
| 2-Oxoglutaric acid | Tricarboxylic acids and derivatives | 115 | 12.182 | 91 | 4.35 |
| cis-Aconitic acid | Tricarboxylic acids and derivatives | 153 | 14.601 | 76 | 4.85 |
| Citric acid | Tricarboxylic acids and derivatives | 143 | 15.433 | 80 | 4.9 |
| Fumaric acid | Tricarboxylic acids and derivatives | 113 | 8.295 | 82 | 5.1 |
| Malic acid | Tricarboxylic acids and derivatives | 103 | 10.437 | 97 | 4.05 |
| Succinic acid | Tricarboxylic acids and derivatives | 115 | 8.154 | 98 | 4.85 |
| 10,12-octadecadienoic acid (C18_2n-10,12c) | Unsaturated fatty acids | 67 | 23.736 | 79 | 5.05 |
| 10-Heptadecenoic acid (C17_1n-7t) | Unsaturated fatty acids | 55 | 21.719 | 97 | 9 |
| 11,14,17-Eicosatrienoic acid (C20_3n-3,6,9c) | Unsaturated fatty acids | 79 | 25.634 | 93 | 6.35 |
| 11,14-Eicosadienoic (C20_2n-6,9c) | Unsaturated fatty acids | 67 | 25.409 | 94 | 6 |
| 13,16-Docosadienoic acid (C22_2n-6,9c) | Unsaturated fatty acids | 67 | 28.569 | 75 | 6.3 |
| Adrenic acid (C22_4n-6,9,12,15c) | Unsaturated fatty acids | 79 | 28.289 | 96 | 6.55 |
| bishomo-gamma-Linolenic acid (C20_3n-6,9,12c) | Unsaturated fatty acids | 79 | 25.278 | 88 | 5.75 |
| Conjugated linoleic acid (C18_2n-9,11c) | Unsaturated fatty acids | 67 | 23.738 | 76 | 5.4 |
| DHA (C22_6n-3,6,9,12,15,18c) | Unsaturated fatty acids | 79 | 28.38 | 98 | 5.6 |
| DPA (C22_5n-3,6,9,12,15c) | Unsaturated fatty acids | 79 | 28.504 | 91 | 5.25 |
| gamma-Linolenic acid (C18_3n-6,9,12c) | Unsaturated fatty acids | 79 | 23.318 | 93 | 6.8 |
| Gondoic acid (C20_1n-9c) | Unsaturated fatty acids | 79 | 25.334 | 71 | 6.15 |
| Linoleic acid (C18_2n-6,9c) | Unsaturated fatty acids | 67 | 23.176 | 94 | 7.1 |

| | | | | | |
|---|---|---|---|---|---|
| Myristoleic acid (C14_1n-5c) | Unsaturated fatty acids | 55 | 16.661 | 75 | 4.3 |
| Nervonic acid (C24_1n-9c) | Unsaturated fatty acids | 55 | 31.236 | 90 | 8.5 |
| Oleic acid (C18_1n-9c) | Unsaturated fatty acids | 55 | 23.154 | 98 | 5.7 |
| Palmitelaidic acid (C16_1n-9c) | Unsaturated fatty acids | 55 | 19.477 | 99 | 6.5 |
| Palmitoleic acid (C16_1n-7c) | Unsaturated fatty acids | 55 | 19.741 | 99 | 6.4 |
| trans-Vaccenic acid | Unsaturated fatty acids | 55 | 23.131 | 98 | 5.75 |

**Supplementary Table S4.** The semi-quantitative log values of identified metabolites in adult giant panda feces, across the six different extraction methods

| Identified metabolite names | Biochemical classes | IPA:ACN:$H_2O$ (3:2:3) (mean +/- SD) | MeOH (100%) (mean +/- SD) | MeOH (80%) (mean +/- SD) | MeOH:$CHCl_3$ (3:1) (mean +/- SD) | ACN (100%) (mean +/- SD) | ACN:$CHCl_3$ (3:1) (mean +/- SD) |
|---|---|---|---|---|---|---|---|
| Dodecane | Alkanes | 4.59±0.01 | 4.64±0.02 | 4.60±0.02 | 4.59±0.02 | 4.60±0.13 | 4.62±0.06 |
| Heptadecane | Alkanes | NA | NA | NA | NA | 4.21±0.16 | 4.20±0.08 |
| Pentadecane | Alkanes | 4.64±0.01 | 4.69±0.01 | 4.66±0.04 | 4.65±0.02 | 4.64±0.14 | 4.63±0.04 |
| Tricosane | Alkanes | NA | NA | NA | 4.36±0.12 | 4.04±0.13 | 4.40±0.05 |
| Tridecane | Alkanes | NA | NA | NA | NA | 4.08±0.14 | 5.10±0.05 |
| beta-Alanine | Amino acid derivatives | 3.81±0.06 | 3.88±0.02 | 3.90±0.03 | 3.74±0.08 | NA | NA |
| N-Acetylglutamic acid | Amino acid derivatives | 3.40±0.04 | NA | NA | NA | NA | NA |
| N-alpha-Acetyllysine | Amino acid derivatives | NA | NA | NA | NA | 3.53±0.10 | 3.66±0.02 |
| Norvaline | Amino acid derivatives | 3.43±0.01 | 3.45±0.06 | 3.45±0.05 | 3.38±0.04 | NA | NA |
| Ornithine | Amino acid derivatives | 4.51±0.08 | 4.22±0.08 | 4.58±0.05 | 4.09±0.09 | 3.34±0.36 | NA |
| Pyroglutamic acid | Amino acid derivatives | 5.09±0.07 | 5.13±0.07 | NA | 4.92±0.12 | NA | NA |
| Alanine | Amino acids | 6.33±0.04 | 6.32±0.02 | 6.35±0.02 | 6.31±0.08 | 4.91±0.02 | 4.77±0.04 |
| Asparagine | Amino acids | 4.55±0.04 | 4.44±0.05 | 4.56±0.01 | 4.33±0.01 | NA | NA |
| Aspartic acid | Amino acids | 5.90±0.09 | 5.83±0.05 | 5.97±0.03 | 5.54±0.12 | 4.06±0.07 | 3.47±0.07 |
| Cysteine | Amino acids | 3.68±0.06 | 3.64±0.04 | 3.82±0.01 | 3.42±0.07 | NA | NA |
| Glutamic acid | Amino acids | 5.51±0.09 | 5.48±0.06 | 5.58±0.01 | 5.27±0.08 | 3.55±0.21 | 3.03±0.07 |
| Glycine | Amino acids | 5.81±0.06 | 5.81±0.03 | 5.86±0.01 | 5.74±0.09 | NA | NA |
| Histidine | Amino acids | 4.57±0.12 | 4.50±0.05 | 4.64±0.04 | 4.48±0.11 | NA | NA |
| Isoleucine | Amino acids | 5.52±0.09 | 5.54±0.04 | 5.60±0.03 | 5.53±0.10 | 4.78±0.09 | 4.86±0.06 |
| Leucine | Amino acids | 6.05±0.09 | 6.06±0.03 | 6.11±0.03 | 6.04±0.10 | 5.28±0.08 | 5.36±0.08 |
| Lysine | Amino acids | 5.64±0.09 | 5.42±0.07 | 5.69±0.03 | 5.31±0.10 | 3.53±0.17 | NA |
| Methionine | Amino acids | NA | 4.45±0.08 | 4.53±0.05 | 4.30±0.10 | 3.22±0.08 | 3.31±0.05 |
| Phenylalanine | Amino acids | 5.61±0.09 | 5.60±0.04 | 5.66±0.02 | 5.58±0.10 | 4.68±0.08 | 4.81±0.07 |
| Proline | Amino acids | 5.98±0.08 | 5.99±0.04 | 6.06±0.03 | 5.98±0.08 | 5.16±0.10 | 5.17±0.07 |
| Serine | Amino acids | 4.07±0.09 | 4.07±0.09 | 4.12±0.08 | 3.93±0.05 | NA | NA |
| Threonine | Amino acids | 4.81±0.08 | 4.73±0.06 | 4.79±0.04 | 4.61±0.09 | NA | NA |
| Tryptophan | Amino acids | 5.51±0.09 | 5.49±0.08 | 5.55±0.06 | 5.49±0.09 | 4.10±0.07 | 4.37±0.06 |
| Tyrosine | Amino acids | 5.63±0.10 | 5.60±0.04 | 5.67±0.05 | 5.58±0.11 | 4.05±0.07 | 4.07±0.04 |
| Valine | Amino acids | 6.00±0.08 | 6.01±0.04 | 6.07±0.03 | 6.00±0.10 | 5.05±0.09 | 5.08±0.05 |
| Benzoic acid | Benzoic acid derivatives | 4.88±0.02 | 4.82±0.03 | 4.86±0.02 | 4.75±0.02 | 4.19±0.12 | 4.19±0.03 |

| | | | | | | | |
|---|---|---|---|---|---|---|---|
| DBP | Benzoic acid derivatives | 4.65±0.01 | 4.63±0.01 | 4.64±0.04 | 4.69±0.01 | 4.62±0.12 | 4.72±0.05 |
| Hydroxybenzoic acid | Benzoic acid derivatives | 5.72±0.06 | 5.67±0.01 | 5.75±0.02 | 5.61±0.02 | 5.37±0.13 | 5.39±0.02 |
| Salicylic acid | Benzoic acid derivatives | 4.28±0.02 | 4.29±0.05 | 4.30±0.01 | 4.25±0.02 | 4.15±0.11 | 4.13±0.04 |
| Syringic acid | Benzoic acid derivatives | 5.55±0.06 | 5.55±0.03 | 5.61±0.03 | 5.47±0.02 | 5.24±0.17 | 5.28±0.01 |
| Vanillic acid | Benzoic acid derivatives | 4.80±0.07 | 4.81±0.05 | 4.87±0.03 | 4.69±0.02 | 4.42±0.14 | 4.43±0.03 |
| 10,13-dimethyltetradecanoic acid (C17_0) | Long-chain saturated FAs | 6.51±0.02 | 6.51±0.01 | 6.47±0.03 | 6.52±0.03 | 6.46±0.14 | 6.53±0.05 |
| Arachidic acid (C20_0) | Long-chain saturated FAs | NA | NA | NA | NA | 4.44±0.11 | 4.56±0.05 |
| Arachidonic acid (C20_4n-6,9,12,15c) | Long-chain saturated FAs | NA | NA | NA | NA | 3.56±0.14 | NA |
| Behenic acid (C22_0) | Long-chain saturated FAs | 4.79±0.06 | 4.88±0.05 | 4.71±0.05 | 4.85±0.07 | 4.47±0.10 | 4.68±0.05 |
| Heneicosanoic acid (C21_0) | Long-chain saturated FAs | NA | 3.95±0.07 | NA | NA | NA | 3.73±0.06 |
| Lignoceric acid (C24_0) | Long-chain saturated FAs | 4.24±0.05 | 4.59±0.11 | 3.82±0.09 | 4.52±0.07 | 3.84±0.13 | 4.34±0.06 |
| Margaric acid (C17_0) | Long-chain saturated FAs | 4.74±0.03 | 4.72±0.02 | 4.70±0.01 | 4.71±0.02 | 4.63±0.12 | 4.70±0.04 |
| Myristic acid (C14_0) | Long-chain saturated FAs | 5.03±0.05 | 5.07±0.03 | 5.01±0.03 | 5.07±0.05 | 5.01±0.14 | 5.05±0.06 |
| Palmitic acid (C16_0) | Long-chain saturated FAs | 4.81±0.01 | NA | NA | NA | NA | NA |
| Pentadecanoic acid (C15_0) | Long-chain saturated FAs | 4.78±0.04 | 4.79±0.02 | 4.74±0.02 | 4.74±0.02 | 4.68±0.12 | 4.74±0.04 |
| Stearic acid (C18_0) | Long-chain saturated FAs | 6.20±0.05 | 6.22±0.02 | 6.09±0.06 | 6.25±0.03 | 6.13±0.13 | 6.27±0.11 |
| Tricosanoic acid (C23_0) | Long-chain saturated FAs | 3.86±0.04 | 4.09±0.11 | 3.65±0.07 | 4.00±0.05 | 3.45±0.12 | 3.78±0.04 |
| 11,14,17-Eicosatrienoic acid (C20_3n-3,6,9c) | Long-Chain unsaturated FAs | NA | NA | 3.48±0.02 | NA | 3.41±0.12 | 3.46±0.04 |
| 11,14-Eicosadienoic (C20_2n-6,9c) | Long-Chain unsaturated FAs | 3.74±0.03 | 3.77±0.04 | 3.74±0.01 | 3.76±0.03 | 3.66±0.14 | 3.74±0.06 |
| Conjugated linoleic acid (C18_2n-9,11c) | Long-Chain unsaturated FAs | 6.26±0.02 | 6.26±0.01 | 6.26±0.02 | 6.24±0.02 | 6.25±0.13 | 6.27±0.04 |
| gamma-Linolenic acid (C18_3n-6,9,12c) | Long-Chain unsaturated FAs | 6.40±0.01 | 6.37±0.02 | 6.39±0.04 | 6.39±0.01 | 6.42±0.14 | 6.41±0.04 |
| Linoleic acid (C18_2n-6,9c) | Long-Chain unsaturated FAs | 4.17±0.08 | NA | 4.22±0.05 | 4.16±0.05 | 4.09±0.11 | 4.16±0.03 |
| Oleic acid (C18_1n-9c) | Long-Chain unsaturated FAs | 5.57±0.01 | 5.54±0.03 | 5.78±0.05 | 5.77±0.01 | NA | 5.56±0.04 |
| Palmitelaidic acid (C16_1n-9c) | Long-Chain unsaturated FAs | 3.87±0.04 | 3.95±0.07 | 3.86±0.03 | 3.82±0.04 | 3.75±0.11 | 3.82±0.04 |
| Palmitoleic acid (C16_1n-7c) | Long-Chain unsaturated FAs | 5.86±0.02 | NA | 5.82±0.03 | 5.87±0.03 | 5.82±0.14 | 5.88±0.05 |
| trans-Vaccenic acid | Long-Chain unsaturated FAs | NA | NA | NA | 5.55±0.02 | 5.51±0.15 | NA |
| 2-Aminoadipic acid | Medium-chain saturated FAs | 3.34±0.06 | 3.37±0.02 | 3.42±0.04 | NA | NA | NA |
| Adipic acid | Medium-chain saturated FAs | NA | NA | NA | NA | 3.63±0.14 | NA |
| Azelaic acid | Medium-chain saturated FAs | 3.94±0.03 | 3.94±0.04 | 3.97±0.03 | 3.82±0.07 | 3.57±0.12 | 3.57±0.03 |

| | | | | | | | |
|---|---|---|---|---|---|---|---|
| Caprinoic acid | Medium-chain saturated FAs | 3.83±0.03 | 4.00±0.02 | 3.84±0.03 | 3.95±0.01 | 3.83±0.14 | 3.78±0.04 |
| Decanoic acid (C10_0) | Medium-chain saturated FAs | 4.32±0.06 | 4.28±0.02 | 4.31±0.02 | 4.23±0.08 | 4.13±0.11 | 4.19±0.03 |
| Dodecanoic acid (C12_0) | Medium-chain saturated FAs | 4.57±0.03 | 4.64±0.02 | 4.50±0.02 | 4.66±0.03 | 4.57±0.15 | 4.54±0.04 |
| Octanoic acid (C8_0) | Medium-chain saturated FAs | 3.83±0.01 | 3.91±0.06 | 3.88±0.05 | 3.85±0.03 | 3.73±0.11 | 3.67±0.03 |
| Suberic acid | Medium-chain saturated FAs | NA | 3.33±0.01 | 3.37±0.05 | 3.25±0.02 | NA | NA |
| Nicotinamide | Nicotinamides and Vitamines | 3.83±0.11 | 3.95±0.06 | 3.93±0.07 | 3.96±0.08 | 3.96±0.12 | 3.87±0.04 |
| Nicotinic acid | Nicotinamides and Vitamines | 3.42±0.05 | 3.34±0.02 | 3.44±0.05 | NA | 5.78±0.08 | NA |
| Creatinine | Secondary metabolites | 4.61±0.18 | 4.60±0.10 | 4.63±0.04 | 4.50±0.12 | 3.80±0.07 | 3.80±0.12 |
| Glutathione | Secondary metabolites | 4.79±0.06 | 4.72±0.04 | 4.85±0.02 | 4.58±0.07 | 3.20±0.06 | NA |
| Lactic acid | Secondary metabolites | 6.71±0.03 | 6.68±0.01 | 6.70±0.01 | 6.68±0.02 | 6.27±0.11 | 6.03±0.08 |
| 2-Oxoglutaric acid | TCA cycle intermediates | 5.26±0.08 | 5.22±0.06 | 5.31±0.05 | 5.14±0.09 | NA | NA |
| cis-Aconitic acid | TCA cycle intermediates | 4.12±0.04 | 3.87±0.01 | 4.12±0.06 | 3.49±0.04 | NA | NA |
| Citric acid | TCA cycle intermediates | 5.00±0.04 | 4.60±0.06 | 4.98±0.03 | 4.08±0.05 | NA | NA |
| Fumaric acid | TCA cycle intermediates | 4.68±0.06 | 4.64±0.03 | 4.70±0.01 | 4.36±0.08 | 3.45±0.01 | NA |
| Malic acid | TCA cycle intermediates | 4.57±0.08 | 4.41±0.05 | 4.65±0.04 | 4.05±0.12 | 3.57±0.06 | NA |
| Succinic acid | TCA cycle intermediates | 6.21±0.08 | 6.18±0.02 | 6.22±0.02 | 6.11±0.09 | 5.21±0.04 | 5.05±0.02 |

**Supplementary Table S5.** The semi-quantitative log values of identified metabolites in baby giant panda feces, across the six different extraction methods

| Identified metabolite names | Biochemical classes | IPA:ACN:$H_2O$ (3:2:3) (mean +/- SD) | MeOH (100%) (mean +/- SD) | MeOH (80%) (mean +/- SD) | MeOH:$CHCl_3$ (3:1) (mean +/- SD) | ACN (100%) (mean +/- SD) | ACN:$CHCl_3$ (3:1) (mean +/- SD) |
|---|---|---|---|---|---|---|---|
| Dodecane | Alkanes | 4.63±0.01 | 4.61±0.01 | 4.60±0.02 | 4.63±0.03 | 4.60±0.05 | 4.63±0.03 |
| Heptadecane | Alkanes | NA | NA | NA | NA | 4.26±0.09 | 4.22±0.02 |
| Pentadecane | Alkanes | 4.67±0.01 | 4.63±0.01 | 4.64±0.03 | 4.64±0.03 | 4.59±0.04 | 4.64±0.02 |
| Tridecane | Alkanes | NA | 4.05±0.05 | 4.09±0.03 | 4.09±0.03 | 4.08±0.05 | 4.08±0.03 |
| beta-Alanine | Amino acid derivatives | 4.14±0.03 | 3.91±0.04 | 4.16±0.02 | 3.85±0.04 | 3.59±0.03 | 3.74±0.02 |
| beta-Citryl-L-glutamic acid | Amino acid derivatives | 3.67±0.08 | NA | NA | NA | NA | NA |
| N-(Carboxymethyl)-L-alanine | Amino acid derivatives | NA | 3.39±0.14 | NA | NA | NA | NA |
| N-Acetylglutamic acid | Amino acid derivatives | 3.72±0.01 | 3.48±0.02 | 3.66±0.04 | 3.47±0.04 | NA | NA |
| N-alpha-Acetyllysine | Amino acid derivatives | NA | 3.71±0.27 | NA | NA | 3.62±0.09 | NA |
| Ornithine | Amino acid derivatives | 4.83±0.05 | 4.19±0.10 | 4.83±0.18 | 4.16±0.03 | NA | NA |
| Pyroglutamic acid | Amino acid derivatives | 4.77±0.01 | 4.48±0.05 | NA | 4.39±0.02 | 3.61±0.07 | 3.72±0.04 |
| Alanine | Amino acids | 5.89±0.03 | 5.68±0.02 | 5.91±0.02 | 5.65±0.03 | 4.34±0.20 | NA |
| Asparagine | Amino acids | 4.04±0.06 | NA | NA | NA | NA | NA |
| Aspartic acid | Amino acids | 5.59±0.06 | 5.23±0.04 | 5.63±0.02 | 5.06±0.03 | 3.63±0.19 | 3.40±0.11 |

| | | | | | | | |
|---|---|---|---|---|---|---|---|
| Cysteine | Amino acids | 4.70±0.07 | 4.11±0.04 | 4.66±0.07 | 4.23±0.02 | 2.84±0.44 | 3.08±0.05 |
| Glutamic acid | Amino acids | 4.90±0.11 | 4.54±0.06 | 4.86±0.02 | 4.51±0.09 | 2.98±0.24 | NA |
| Glycine | Amino acids | 5.63±0.01 | 5.36±0.02 | 5.65±0.03 | 5.26±0.03 | NA | NA |
| Histidine | Amino acids | 4.46±0.03 | NA | 4.45±0.11 | NA | NA | NA |
| Isoleucine | Amino acids | 5.48±0.02 | 5.35±0.03 | 5.51±0.03 | 5.31±0.03 | 4.21±0.16 | 4.44±0.04 |
| Leucine | Amino acids | 5.79±0.01 | 5.58±0.01 | 5.76±0.02 | 5.57±0.03 | 4.49±0.14 | 4.72±0.02 |
| Lysine | Amino acids | 5.29±0.07 | 4.56±0.07 | 5.18±0.08 | 4.58±0.03 | NA | NA |
| Methionine | Amino acids | 3.82±0.22 | 4.04±0.04 | 4.26±0.09 | 3.79±0.08 | 2.83±0.08 | NA |
| Phenylalanine | Amino acids | 5.26±0.01 | 5.05±0.01 | 5.24±0.03 | 5.02±0.02 | 3.91±0.16 | 4.11±0.02 |
| Proline | Amino acids | 5.67±0.01 | 5.49±0.01 | 5.70±0.02 | 5.46±0.03 | 4.49±0.08 | 4.59±0.01 |
| Serine | Amino acids | 4.11±0.05 | 3.65±0.13 | 4.01±0.11 | 3.67±0.05 | NA | NA |
| Threonine | Amino acids | 4.86±0.01 | 4.37±0.07 | 4.75±0.06 | 4.39±0.05 | NA | NA |
| Tryptophan | Amino acids | 6.04±0.03 | 5.76±0.01 | 6.00±0.04 | 5.70±0.03 | 3.94±0.31 | 4.52±0.05 |
| Tyrosine | Amino acids | 4.81±0.03 | 4.44±0.01 | 4.72±0.08 | 4.43±0.05 | 2.93±0.26 | NA |
| Valine | Amino acids | 5.94±0.01 | 5.75±0.02 | 5.94±0.02 | 5.71±0.02 | 4.52±0.16 | 4.66±0.03 |
| Benzoic acid | Benzoic acid derivatives | 4.03±0.01 | 4.15±0.02 | 3.95±0.03 | NA | 4.00±0.05 | 4.18±0.05 |
| DBP | Benzoic acid derivatives | 4.63±0.03 | 4.57±0.01 | 4.62±0.03 | 4.62±0.02 | 4.51±0.04 | 4.58±0.02 |
| Hydroxybenzoic acid | Benzoic acid derivatives | 4.07±0.01 | NA | 4.08±0.04 | NA | NA | NA |
| Salicylic acid | Benzoic acid derivatives | NA | NA | 3.37±0.86 | NA | NA | NA |
| Adipic acid | Medium-chain saturated FAs | 3.94±0.03 | 3.81±0.04 | 3.94±0.10 | 3.82±0.02 | 3.55±0.02 | 3.68±0.05 |
| Azelaic acid | Medium-chain saturated FAs | 3.47±0.04 | 3.25±0.05 | NA | 3.35±0.06 | 3.10±0.05 | 3.21±0.03 |
| Decanoic acid (C10_0) | Medium-chain saturated FAs | 4.30±0.04 | 3.99±0.01 | 4.10±0.02 | 4.10±0.02 | 3.74±0.07 | 3.88±0.05 |
| Dodecanoic acid (C12_0) | Medium-chain saturated FAs | 5.77±0.03 | 5.47±0.06 | 5.57±0.03 | 5.54±0.06 | 5.41±0.07 | 5.55±0.10 |
| Hexanoic acid (C6_0) | Medium-chain saturated FAs | 4.03±0.06 | 3.67±0.07 | 3.76±0.06 | 3.98±0.08 | NA | NA |
| Octanoic acid (C8_0) | Medium-chain saturated FAs | 3.79±0.04 | 3.60±0.03 | 3.64±0.05 | 3.61±0.06 | 3.40±0.05 | 3.47±0.04 |
| Suberic acid | Medium-chain saturated FAs | 4.43±0.03 | 3.70±0.04 | 3.90±0.05 | 3.74±0.04 | 5.43±0.06 | 3.46±0.02 |
| Nicotinamide | Nicotinamides and Vitamines | 3.84±0.03 | 3.77±0.02 | 3.79±0.04 | 3.82±0.06 | 3.79±0.06 | 3.81±0.02 |
| 10,13-dimethyltetradecanoic acid (C17_0) | Long-chain saturated FAs | 6.81±0.01 | 6.77±0.02 | 6.78±0.02 | 6.78±0.02 | 6.75±0.05 | 6.77±0.01 |
| 2-Methyloctadecanoic acid | Long-chain saturated FAs | NA | NA | NA | NA | 3.75±0.15 | 4.19±0.14 |
| Arachidic acid (C20_0) | Long-chain saturated FAs | 5.22±0.01 | 5.13±0.05 | 4.90±0.04 | 5.20±0.05 | 4.89±0.05 | 5.04±0.04 |
| Arachidonic acid (C20_4n-6,9,12,15c) | Long-chain saturated FAs | 6.17±0.01 | 6.15±0.02 | 6.12±0.03 | 6.13±0.01 | 5.72±0.06 | 5.93±0.04 |
| Behenic acid (C22_0) | Long-chain saturated FAs | 5.00±0.02 | 5.00±0.06 | 4.39±0.01 | 5.07±0.04 | 4.70±0.05 | 4.94±0.06 |
| Heneicosanoic acid (C21_0) | Long-chain saturated FAs | 3.96±0.03 | 3.91±0.04 | NA | 3.96±0.05 | 3.49±0.07 | 3.73±0.03 |
| Lignoceric acid (C24_0) | Long-chain saturated FAs | 4.58±0.02 | 4.75±0.05 | NA | 4.87±0.03 | 4.20±0.06 | 4.63±0.06 |
| Margaric acid (C17_0) | Long-chain saturated FAs | 5.13±0.02 | 4.99±0.06 | 4.94±0.04 | 5.03±0.04 | 4.73±0.07 | 4.86±0.05 |
| Myristic acid (C14_0) | Long-chain saturated FAs | 6.46±0.02 | 6.32±0.04 | 6.34±0.02 | 6.34±0.04 | 6.21±0.06 | 6.30±0.06 |

| | | | | | | | |
|---|---|---|---|---|---|---|---|
| Nonadecanoic acid (C19_0) | Long-chain saturated FAs | 4.84±0.01 | 4.80±0.02 | 4.61±0.07 | 4.83±0.03 | 4.06±0.07 | 4.29±0.05 |
| Pentadecanoic acid (C15_0) | Long-chain saturated FAs | 5.24±0.02 | 5.11±0.03 | 5.12±0.03 | 5.14±0.03 | 4.87±0.08 | 5.01±0.04 |
| Stearic acid (C18_0) | Long-chain saturated FAs | 6.79±0.01 | 6.77±0.02 | 6.74±0.02 | 6.78±0.02 | 6.70±0.04 | 6.73±0.01 |
| Tricosanoic acid (C23_0) | Long-chain saturated FAs | 4.04±0.04 | 4.10±0.07 | NA | 4.25±0.05 | 3.60±0.04 | 3.99±0.04 |
| Creatinine | Secondary metabolites | 4.53±0.07 | 4.35±0.06 | 4.51±0.07 | 4.28±0.08 | 3.13±0.10 | 3.54±0.05 |
| Glutathione | Secondary metabolites | 4.12±0.03 | 3.92±0.04 | 4.17±0.01 | 3.75±0.06 | #NUM! | NA |
| Lactic acid | Secondary metabolites | 5.81±0.01 | 5.51±0.04 | 5.76±0.02 | 5.47±0.05 | 4.38±0.13 | 4.35±0.09 |
| 2-Oxoglutaric acid | TCA cycle intermediates | 3.91±0.06 | 3.60±0.03 | 3.85±0.05 | 3.59±0.04 | 4.40±0.13 | NA |
| cis-Aconitic acid | TCA cycle intermediates | 3.63±0.03 | 3.46±0.06 | 3.71±0.03 | 3.39±0.05 | NA | NA |
| Citric acid | TCA cycle intermediates | 3.93±0.05 | NA | 3.83±0.06 | NA | NA | NA |
| Fumaric acid | TCA cycle intermediates | 3.88±0.03 | 3.61±0.06 | 3.73±0.01 | 3.61±0.02 | NA | NA |
| Malic acid | TCA cycle intermediates | 3.94±0.03 | 3.61±0.07 | 3.67±0.05 | 3.84±0.19 | 3.33±0.10 | 3.39±0.14 |
| Succinic acid | TCA cycle intermediates | 6.09±0.02 | 5.94±0.04 | 6.05±0.02 | 5.92±0.03 | 5.42±0.07 | 5.82±0.05 |
| 10,12-octadecadienoic acid (C18_2n-10,12c) | Long-chain unsaturated FAs | NA | NA | 3.99±0.05 | NA | NA | NA |
| 10-Heptadecenoic acid (C17_1n-7t) | Long-chain unsaturated FAs | 5.14±0.03 | 5.13±0.04 | 5.10±0.06 | 5.14±0.03 | 4.49±0.07 | 4.75±0.04 |
| 11,14,17-Eicosatrienoic acid (C20_3n-3,6,9c) | Long-chain unsaturated FAs | 4.79±0.02 | 4.73±0.03 | 4.69±0.05 | 4.74±0.03 | 4.27±0.07 | 4.48±0.05 |
| 11,14-Eicosadienoic (C20_2n-6,9c) | Long-chain unsaturated FAs | 5.26±0.01 | 5.22±0.02 | 5.15±0.04 | 5.24±0.01 | 4.76±0.07 | 4.98±0.05 |
| 13,16-Docosadienoic acid (C22_2n-6,9c) | Long-chain unsaturated FAs | 4.33±0.01 | 4.38±0.02 | NA | 4.36±0.03 | 3.83±0.07 | 4.12±0.07 |
| Adrenic acid (C22_4n-6,9,12,15c) | Long-chain unsaturated FAs | 4.93±0.02 | 4.86±0.04 | 4.79±0.05 | 4.90±0.02 | 4.16±0.06 | 4.53±0.05 |
| bishomo-gamma-Linolenic acid (C20_3n-6,9,12c) | Long-chain unsaturated FAs | 5.81±0.01 | 5.80±0.01 | 5.74±0.02 | 5.79±0.01 | 5.35±0.07 | 5.55±0.05 |
| Conjugated linoleic acid (C18_2n-9,11c) | Long-chain unsaturated FAs | 4.05±0.03 | 3.95±0.02 | NA | 4.02±0.05 | 3.57±0.05 | 3.76±0.03 |
| DHA (C22_6n-3,6,9,12,15,18c) | Long-chain unsaturated FAs | 5.72±0.01 | 5.65±0.04 | 5.63±0.03 | 5.64±0.02 | 5.08±0.06 | 5.37±0.05 |
| DPA (C22_5n-3,6,9,12,15c) | Long-chain unsaturated FAs | 5.34±0.01 | 5.29±0.04 | 5.23±0.03 | 5.28±0.01 | 4.67±0.05 | 5.00±0.06 |
| gamma-Linolenic acid (C18_3n-6,9,12c) | Long-chain unsaturated FAs | NA | NA | 5.08±0.03 | NA | 4.17±0.05 | 4.97±0.04 |
| Gondoic acid (C20_1n-9c) | Long-chain unsaturated FAs | 5.24±0.06 | 5.43±0.05 | 5.33±0.10 | 5.46±0.06 | 4.76±0.05 | 4.98±0.09 |
| Linoleic acid (C18_2n-6,9c) | Long-chain unsaturated FAs | 6.21±0.02 | 6.20±0.02 | 6.19±0.03 | 6.20±0.02 | 5.90±0.06 | 6.04±0.03 |
| Myristoleic acid (C14_1n-5c) | Long-chain unsaturated FAs | 3.83±0.01 | 3.71±0.02 | 3.78±0.02 | 3.76±0.01 | NA | NA |
| Nervonic acid (C24_1n-9c) | Long-chain unsaturated FAs | 4.82±0.01 | 4.82±0.08 | 4.34±0.07 | 4.88±0.04 | 4.25±0.05 | 4.66±0.06 |

| | | | | | | | |
|---|---|---|---|---|---|---|---|
| Oleic acid (C18_1n-9c) | Long-chain unsaturated FAs | 6.18±0.01 | NA | 6.15±0.02 | 6.16±0.02 | 5.85±0.05 | 6.14±0.01 |
| Palmitelaidic acid (C16_1n-9c) | Long-chain unsaturated FAs | 5.09±0.02 | 5.06±0.02 | 5.06±0.03 | 5.06±0.03 | 4.64±0.07 | 4.83±0.05 |
| trans-Vaccenic acid | Long-chain unsaturated FAs | NA | 6.16±0.02 | NA | NA | 5.93±0.06 | 6.04±0.03 |

Supplementary Table S6. The total amount of estimated time for sample preparation, derivatization, and GC-MS analysis per sample.

| **Steps** | **Descriptions** | **Duration** |
|---|---|---|
| Sample Preparation | Grind sample with pestle and mortar | 2 min |
| | Weight sample | 0.5 min |
| | Add extraction solvents | 1 min |
| | Homogenize by Tissuelyser | 3 min |
| | Centrifugation | 15 min |
| | Dry samples by SpeedVac | 180 min |
| Derivatization | Time for MCF reaction time | 1 min |
| | Add reagent | 1 min |
| | Vortex | 0.1 min |
| | Centrifugation | 5 min |
| | Extract metabolites from chloroform phase | 1 min |
| GC-MS analysis | Inject sample and wish syringe | 3 min |
| | GC-MS analysis time | 43 min |
| | **Total Time:** | 255.6 min |
